# The Challenges of Bringing Up a Child with Autism Spectrum Disorders: Editorial for Brain Sciences Special Issue “Connections between Parental Involvement and Treatment of Children with Autism Spectrum Disorders (ASD)”

**DOI:** 10.3390/brainsci11050618

**Published:** 2021-05-12

**Authors:** Sayyed Ali Samadi

**Affiliations:** Centre for Intellectual and Developmental Disabilities-Institute of Nursing Research, University of Ulster, Newtownabbey BT37 0QB, Northern Ireland, UK; s.samadi@ulster.ac.uk

There is no unique scientific method to guide parents in bringing up a child with autism spectrum disorders (ASD). Likewise, there are no distinctive interventions that can meet all the needs of individuals with ASD. Therefore, the aim of this Special Issue was not to focus on one particular model of intervention. Rather, the goal was to illustrate how the role of parents can be taken more seriously in whatever interventions are used or under development.

The papers in this Special Issue explore the transitioning and passing on of new styles of programs to parents and the community. This involves a conceptual shift where caregivers are considered equal members of the professional treatment team for the child. This means going beyond considering parents’ opinions and receiving their support, enlisting the active participation of parents in training sessions and clinical decision-making in identifying goals and opportunities for the children, as well as in generalizing and maintaining newly acquired skills for themselves and other family members.

Underpinning this changed approach with parents is the adoption of new ways of thinking about ASD. In the main, ASD is broadly described as a medical diagnosis based on DSM or ICD definitions of behaviors and clinical symptoms. Hence, a medical model of service provision has been adopted in designing intervention goals. The medical model assumes that the curing or managing of a disability, either generally or completely, revolves around identifying the illness or disability from the in-depth clinical perspective of the individual. Yet, modern conceptions of disabilities—including ASD—acknowledge the impact of environmental influences on a child’s development, such as the role of parents and family interactions as well as community and societal influences. Together, these can have strong influences on the levels of function of the individual with different types of disabilities [1].

This new perspective makes new space for parental engagement in the process of professional support for children with developmental disabilities in both assessment and intervention. The International Classification of Functioning, Disability and Health [2] describes a new assessment framework that considers the social and environmental impacts on medical and disabling conditions. This classification compares the person’s current level of performance and the barriers they experience with the actions needed to enhance their functioning in their surrounding environments of the family and community. The adoption of social models may be particularly crucial in redefining the parental impacts on service provision and understanding their involvement in the intervention and treatment process for children with ASD. This has led to the creation of family-centered interventions for children with additional needs, be they through illness or disability.

Family-centered interventions is a general term that covers both a philosophy and a method of service delivery. It consists of a composition of elements such as values, skills, behaviors, and knowledge that recognizes the centrality of parents in the lives of their child with developmental disabilities. The main endeavor is to consider the unique position of every family member in supporting an individual with developmental disabilities to learn, grow, and thrive. It puts parental values, natural lifestyle, and the strengths, needs, and ideas of people with a disability and their parents and caregivers as the focal point of service provision, including the design of interventions and their implementation and evaluation.

Dunst et al. [3] demonstrated the efficacy of family-centered practice with all types of developmental programs. However, this approach was less developed in ASD at that time. While studies on parent-mediated ASD intervention have evolved gradually, available results indicate that more research is needed to understand the full impact of parent-mediated interventions on the child and family [4].

Parental efficacy plays a crucial role in family-centered practice, as it is also linked to family functioning in general. Family functioning theories emphasize the primary role of parents as the main component of the family unit, in that they provide the basis for the development and maintenance of all family members biologically, socially, and psychologically. Understanding parental and family members’ needs for development in these different aspects is essential in order to facilitate the family’s role in caregiving for a child with ASD. Enhancing parental awareness about their impacts, authorities, and roles can be obtained through different sources, such as training opportunities and listening to their voice when they talk about their ideas or explain issues from their perspective.

In this Special Issue, different contributing factors of parental involvement in the treatment of children with ASD have been considered. The coverage extends to the parental role in contributing to the general health and development of their offspring in other socially vulnerable groups as well as parents of children with ASD. Nevertheless, different aspects were not fully covered, and there is still much to learn. Paternal involvement in the treatment process of children with ASD is still in its early stages of progress. There is also a dearth of cross-cultural studies concerning parental involvement. A new area of research might be the impact of online diagnosis and intervention services that inevitably have to consider parents as the key person in creating these new styles of services. Profound changes due to the COVID-19 pandemic have impacted how intervention and assessment services are carried out by therapists, clinicians, and researchers. Research into parental satisfaction with these services and the effective provision of help and support to them could transform current provisions and usher in a new era of partnership working between professionals and parents [5].

In summation, it has been my privilege to work with the contributors, reviewers, and editors on this Special Issue. I appreciate their kind assistance, as it would not be possible to attain our goal without their time and consideration.
